# Sleep disorders among medical students: Prevalence and associated factors

**DOI:** 10.1192/j.eurpsy.2026.12103

**Published:** 2026-07-31

**Authors:** S. Bradai, N. Ketata, H. Ben ayed, M. Ben Hamida, M. Kassis, J. Jedidi, S. Yaich

**Affiliations:** 1 Hedi Chaker hospital; 2 Habib bourguiba hospital; 3 faculty of medicine of sfax, sfax, Tunisia

## Abstract

**Introduction:**

Sleep disorders are highly prevalent worldwide, and medical students (MS) are particularly vulnerable because of the considerable academic pressure they face.

**Objectives:**

This study aimed to investigate the prevalence of insomnia among medical students and to identify their associated factors

**Methods:**

A cross-sectional study was conducted among a randomized sample of MS in Sfax, Southern Tunisia, during the Academic year 2024-2025. Data collection was performed using an anonymous self-administered questionnaire including the validated *Insomnia Severity Index.* A cut-off score of 8 or higher was adopted to determine the prevalence of insomnia among participants

**Results:**

A total of 73 MS were eligible for the study. The median age was 21 years with an interquartile range (IQR)= [20-23,5 years]. There were 41 females (56.2%), giving a male to female ratio of 1.28. History of chronic disease was noted among 14 MS (19.1%) and 41 MS had neck pain (56.2%). As for lifestyle behaviours, 23 MS (31.5%) were smokers and 8 MS (11%) practised sports.

Prevalence of Insomnia among MS was 60.3% (n=44), of whom, 3 MS (6.18%) had Severe insomnia.

Insomnia was statistically associated with having chronic disease (OR=2,1; p=0,04), having neck pain (OR=1.951; p=0.011), depression (OR=1.715; p=<0.001), anxiety (OR=1.696; p=0.025) and stress (OR 1.409*;* p=0.013). Besides, practicing sports was statistically associated with lower prevalence of insomnia among MS (OR=0.21; P=0.034).

**Conclusions:**

A high prevalence of Insomnia was observed among MS, with multiple psychological and lifestyle factors associated with insomnia. These findings highlighted the need to prioritize mental health interventions to promote well-being and improve sleep quality in this vulnerable population.

**Disclosure of Interest:**

None Declared

